# Health and income inequality: a comparative analysis of USA and Italy

**DOI:** 10.3389/fpubh.2024.1421509

**Published:** 2024-08-07

**Authors:** Caterina A. M. La Porta, Stefano Zapperi

**Affiliations:** ^1^Center for Complexity and Biosystems and Center for Innovation for Well-Being And Environment, Department of Environmental Science and Policy, University of Milan, Milan, Italy; ^2^UOC Maxillo-Facial Surgery and Dentistry, Fondazione IRCCS Ca' Granda Ospedale Maggiore Policlinico, Milan, Italy; ^3^Center for Complexity and Biosystems, Department of Physics “Aldo Pontremoli”, University of Milan, Milan, Italy; ^4^CNR—Consiglio Nazionale delle Ricerche, Istituto di Chimica della Materia Condensata e di Tecnologie per l'Energia, Milan, Italy

**Keywords:** health inequality, comorbidity, income inequality, social mobility, obesity, food insecurity

## Abstract

**Introduction:**

Socio-economic background is often an important determinant for health with low income households having higher exposure to risk factors and diminished access to healthcare and prevention, in a way that is specific to each country.

**Methods:**

Here, we perform a comparative analysis of the relations between health and income inequality in two developed countries, USA and Italy, using longitudinal and cross-sectional data from surveys.

**Results and discussion:**

We show that the income class determines the incidence of chronic pathologies, associated risk-factors and psychiatric conditions, but find striking differences in health inequality between the two countries. We then focus our attention on a fraction of very disadvantaged households in the USA whose income in persistently at the bottom of the distribution over a span of 20 years and which is shown to display particularly dire health conditions. Low income people in the USA also display comorbidity patterns that are not found in higher income people, while in Italy income appears to be less relevant for comorbidity. Taken together our findings illustrate how differences in lifestyle and the healthcare systems affect health inequality.

## 1 Introduction

The concept of human health has changed over time since the definition given in 1948 by the WHO and expanded in 1986 (1), including not only health and diseases but the general concept of wellbeing. Health and wellbeing are, however, not equally distributed throughout the human population as shown by greatest lifespan variations across countries, with less developed countries showing shorter lifespans and higher mean mortality rates (2). The relation between health disparities and income inequality within an individual country has been well-documented in the literature (3, 4). Health inequality implies that disadvantaged groups within the population face higher disease rates also due limitations in the access to healthcare facilities (5). Among Western countries, the USA displays remarkable associations between life expectancy and income (6): A recent study showed that people belonging to the top 1% of the income distribution had a life expectancy that was more than 10 years longer than that expected for people in the bottom 1% of the distribution (6). There have been several attempts to link health inequalities to sociodemographic characteristics, such as sex, race, geography (7) or educational attainment (8), to biological factors (9) and behavioral aspects (10). Pickett and Wilkinson (11) presented an in depth review of the literature to establish a causal relationship between income inequality and health from an epidemiological perspective. They concluded that income inequality has a causal effect on health and wellbeing according to a set of standard causal criteria. Furthermore, Thomson et al. (12) conducted a systematic review demonstrating the causal association between income inequality and mental health conditions.

Health inequality in Western Europe appears generally to follow different path than in the USA, since overall mortality is declining in Europe with little dependence on income or education. Despite this generally positive outlook, recent economic crises created a decrease in the health conditions and an increase in stress and anxiety, but were generally not associated with a widening of health inequality across Europe (13). The mortality decline in Europe has been explained by the constant improvements in living standards and in disease prevention and care that were able to offset the negative effects of the economic crisis (13). Furthermore, there has been a decline in the exposure to some important risk factors such as smoking. In most recent years, life expectancy has seen a rapid decline that can mostly be attributed to the COVID-19 pandemic (14).

In this paper, we compare health inequalities in two developed countries, USA and Italy, that display important differences in terms of lifestyle and access to healthcare. The USA has been for decades the largest world economy known for its immense wealth, large average income and high living standards. Italy, while also an industralized country, is a smaller economy with lower average income and wealth. Contrary to the USA, where healthcare and retirement benefits are mostly provided by employment-based private health insurances and retirement plans, Italy has a more extensive public social welfare system which provides universal healthcare and pensions. In a previous study (15), we compared income and wealth inequalities in these two countries showing that the USA displays wider inequalities in wealth and income than Italy. By analyzing in detail the mobility of households across the wealth distribution, we also found that in both countries low and high wealth households are generally the most immobile (15). Our previous work highlighted that the wider economic inequality observed in the USA was also associated with poorer performance in health status compared to Italy, as revealed by macro-level indicators such as disease-related mortality (15). Here, we perform a statistical analysis of national survey data to correlate in more details income level and health conditions across the populations of these two countries.

## 2 Methods

### 2.1 Data

To study changes in income and health in the USA through longitudinal data, we downloaded data from the Panel Study of Income Dynamics (PSID) (16), public use dataset, produced and distributed by the Survey Research Center, Institute for Social Research, University of Michigan, Ann Arbor, MI (last accessed on 23/03/2024 from: https://psidonline.isr.umich.edu/) Cross-sectional data on income and health for Italy was obtained from the European Health Interview Survey (EHIS) conducted by the Italian National Institute of Statistics (ISTAT) last accessed on on 20/03/2024 from: https://www.istat.it/en/archivio/214912. Longitudinal income data for Italy was obtained from the Bank of Italy (BoI) for the years 1991–2020 (“Indagine sui bilanci delle famiglie italiane”) last accessed on 22/3/2023 from: https://www.bancaditalia.it/statistiche/basi-dati/rdc/index.html. Aggregated statistical series for life expectancy were obtained from the World Bank (WB) database, last accessed on 20/03/2024 at: https://data.worldbank.org/. Dietary compositions for USA and Italy was obtained from the Food and Agriculture Organization of the United Nations (FAO) at: https://www.fao.org/faostat/en/#home last accessed on 26/03/2024. Data on obesity in the USA were obtained from the Behavioral Risk Factor Surveillance System (BRFSS) of the Centers for Disease Control and Prevention (CDC; https://www.cdc.gov/brfss/data_documentation/index.htm).

### 2.2 Income distribution

To estimate the income distribution, we employed logarithmically spaced bins following La Porta and Zapperi (15). This is justified by the fact that the income distribution displays power-law (or Pareto) tails that would be misrepresented by more conventional linearly-spaced bins. Given the income *Y*_*i*_(*t*) of household *i* at time *t*, we first normalized the variable by its weighted mean as *y*_*i*_ = *Y*_*i*_/〈*Y*_*i*_〉, where $\langle Y_{i}\rangle =\underset{i}{\sum }p_{i}Y_{i}$. The normalized weights $p_{i}=M_{i}/\underset{i}{\sum }M$ take into account the statistical weights *M*_*i*_ associated to each household. We selected the positive values *Y*_*i*_>0 and log-transform the normalized variable *X*_*i*_ = log_10_(*Y*_*i*_/〈*Y*_*i*_〉). We then construct the histogram of *X*_*i*_ by defining a set of linearly-spaced intervals summing the statistical weights of the data points falling in each bin. The resulting counts in each interval were then divided by the length of the interval.

### 2.3 Income quintiles

In our analysis, we classified each household according to the quintile interval to which it belongs within the income distribution (15). Working with quantiles is useful when comparing income distributions over different years because it suppresses any biases due to inflation and allowed us to focus on relative income. Given the income *Y* whose cumulative distribution is *F*(*Y*), we defined quintiles *Y*_*q*_ as *F*(*Y*_*q*_) = *q*/5 for *q* = 0, ..., 4. The quintile intervals were then defined as [*Y*_0_, *Y*_1_], ... [*Y*_3_, *Y*_4_], [*Y*_4_, ∞]. For simplicity, we denote the quintile interval [*Y*_*q*_, *Y*_*q*+1_] by *q*. In practice, we sorted households according to their wealth and divided them in 5 intervals of equal weight. Thus, to each data point *Y*_*i*_ we associated a number 0 ≤ *q*_*i*_ ≤ 4, where *q* = 0 represents the bottom 20% of the income distribution and *q* = 4 the top 20%. We refer to this number as the income quintile *q*_*i*_ of household *i*.

### 2.4 Transition matrices and persistence

We studied the dynamics *q*_*i*_(*t*) of the income quintiles associated to each household by following its time evolution for the time span available in the longitudinal surveys. For the sake of comparison, we restrict the analysis to a time span of 20 years. From the quintile trajectories *q*_(_*t*), we estimated transition probability matrices


(1) $$\begin{array}{l} P_{Q}\left(q^{\prime },t|q,t_{0}\right)=P\left(q_{i}\left(t\right)=q^{\prime }|q_{i}\left(t_{0}\right)=q\right) \end{array}$$

describing the probability that $q_{i}=q^{\prime }$ at time *t* when *q*_*i*_ = *q* at time *t*_0_. The time-dependent persistence relative to the income quintile *q* is defined as the probability to remain in the initial quintile at time *t*


(2) $$\begin{array}{l} \Phi_{Q}\left(t|q,t_{0}\right)=P_{Q}\left(q,t|q,t_{0}\right). \end{array}$$

## 3 Results

### 3.1 Income inequality and mobility in Italy and USA

**Figure 1:**
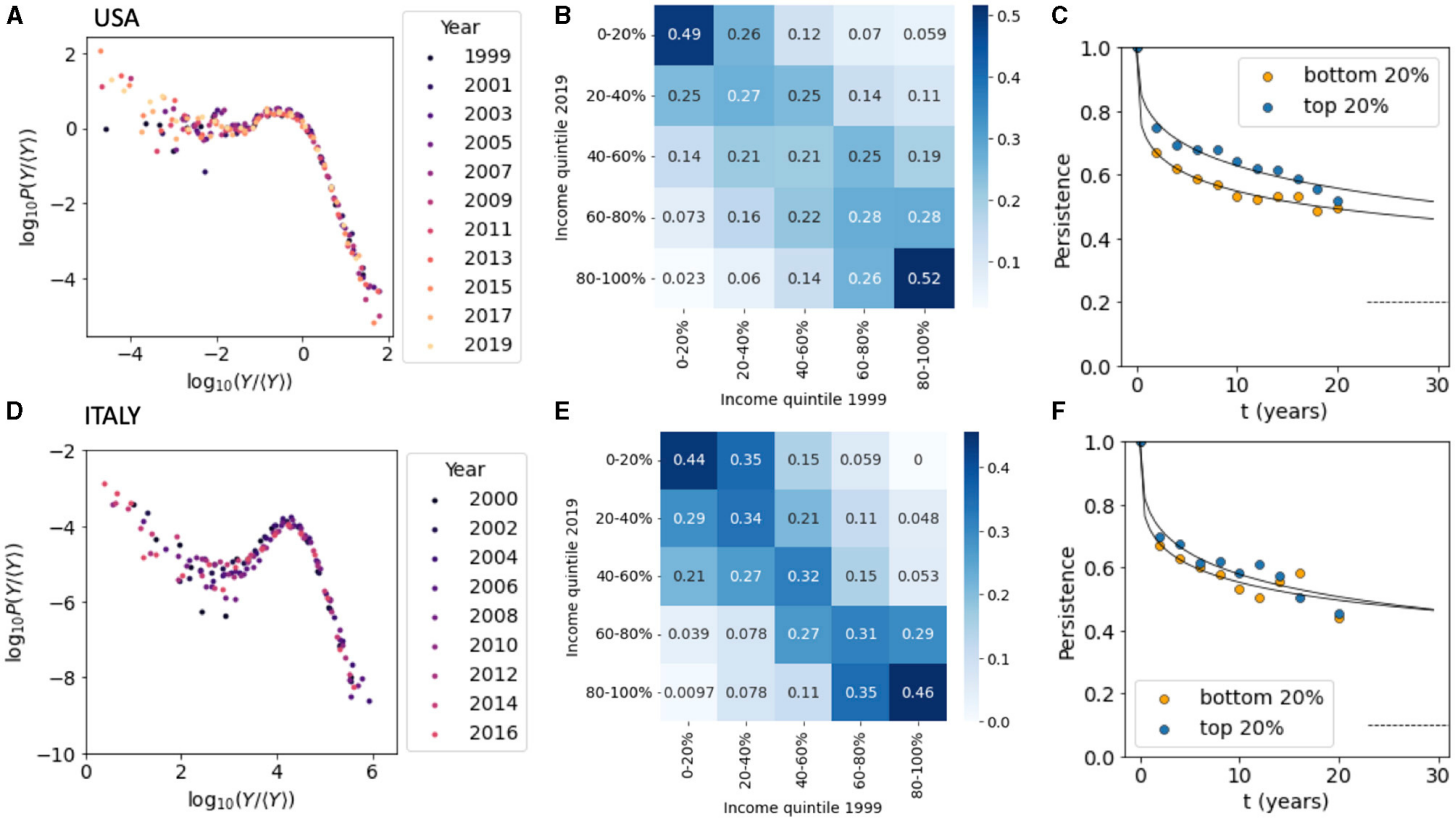
Income inequality in USA and Italy. **(A)** The distribution of the relative income for households in the USA from 1999 to 2019. **(B)** The transition matrix among income quintile classes from 1999 to 2019 for the USA. **(C)** The persistence for households in the top and bottom 20% of the income distribution for US household. **(D)** Same as **(A)** but for Italy. **(E)** Same as **(B)** but for Italy. **(F)** Same as **(C)** but for Italy. Data sources: **(A–C)** PSID, **(D–F)** ISTAT-EIHS.

We studied the patterns of income inequality and the mobility among income classes for households in the USA and Italy over a period of 20 years. To quantify income inequality, we estimated the probability density function of the relative income, normalized by the average income, for different years. The results shown in Figure 1A for the USA shows that the shape of the distribution remained relatively stable over the study period. The distribution displays the characteristic power-law Pareto tail for high income values, scaling as ρ(*x*)~*x*^−(1+α)^ with α≃2. The distribution deviates from a power law for income values below the mean.

We next considered the transition matrix between different income classes by dividing the income distribution in quintiles and calculating the probability that a households belonging to quintile *q* at *t*_0_ = 1999 is found in quintile *q*′ at *t* = 2019 (Equation 1). The matrix depicted in Figure 1B for USA displays the form typically observed in intergenerational transition matrices for income and wealth, with households found in the upper and lower quintiles that are more likely to remain there after two decades (17, 18). Following La Porta and Zapperi (15), we focused then on households persistence (Equation 2) in the top and bottom income quintile and followed their time dependence (Figure 1C). We can see that after a rapid drop the persistence decays very slowly and is well-described by a stretched exponential relaxation (15). In practice, about a half of the households that were part of the top and bottom 20% of the income distribution remain in the same quintile after 20 years (as also indicated in Figure 1B).

The same analysis was then performed for Italian households for years from 2000 to 2020. The income distribution follows the same pattern as the US one, with a power law tail for incomes larger than the mean. The Pareto exponent is found to be larger than in the USA and can be estimated as α≃2.7, indicating that the income distribution is more unequal in the USA than it is in Italy (Figure 1D). The mobility matrix for Italy, on the other hand, is very close to the one measured in the USA (Figure 1E, see also Acciari et al. (19) for a recent study of intergenerational income mobility in Italy) with a persistence in the top and bottom income quintiles decreasing slowly in time (Figure 1F).

### 3.2 Income inequality and health

**Figure 2:**
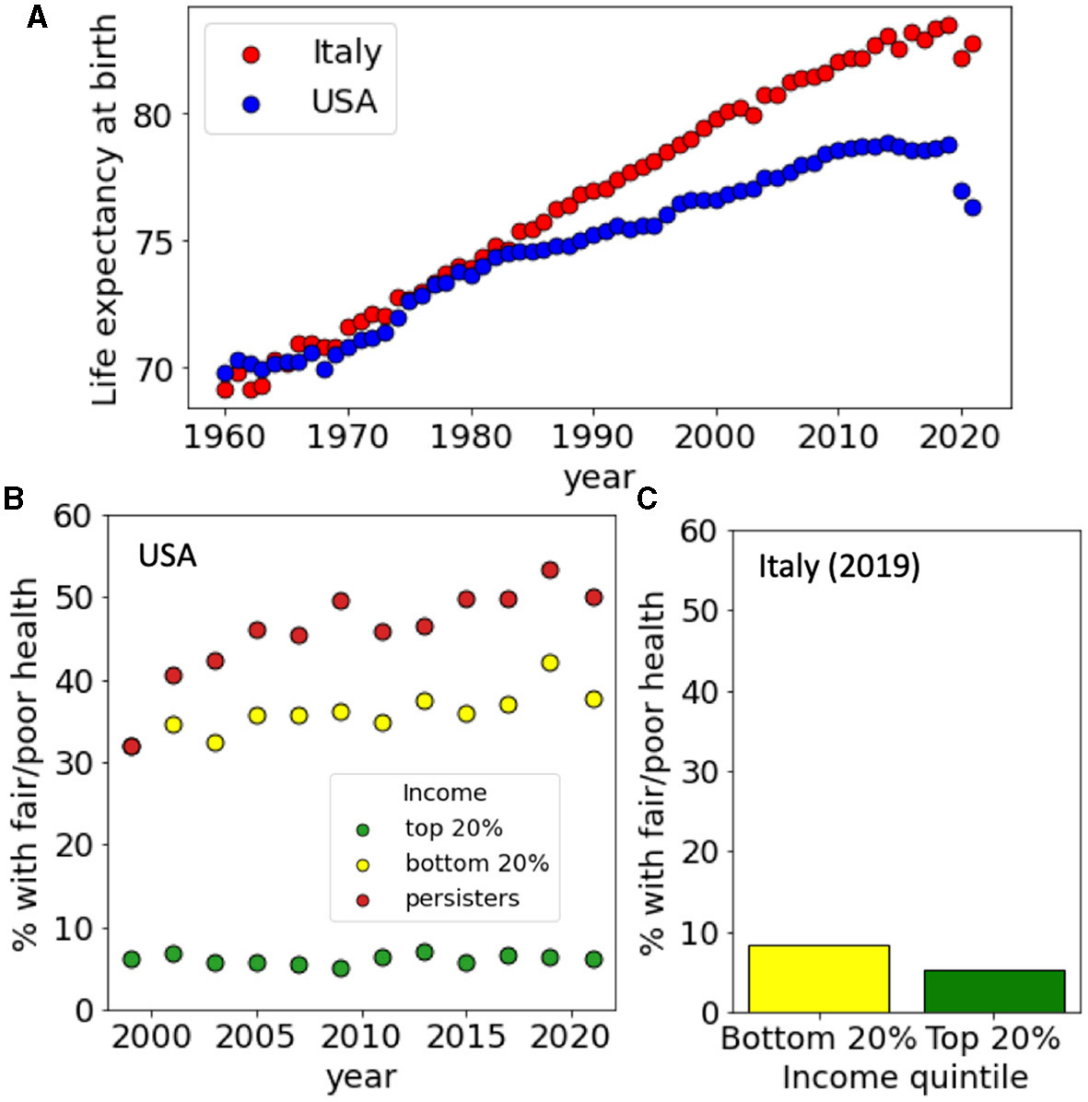
Life expectancy and income-dependence of the health status in USA and Italy. **(A)** Life expectancy at birth for USA and Italy from 1960 to 2020. **(B)** Fraction of people with poor or fair health for US households belonging to the top and bottom 20% of the income distribution and for persisters from 1999 to 2021. **(C)** Fraction of people with poor or fair health for Italian households belonging to the top and bottom 20% of the income distribution in 2019. Data sources: **(A)** World Bank, **(B)** PSID, and **(C)** ISTAT-EIHS.

The life expectancy at birth of the Italian and US populations were similar in 1960 but started to diverge around 1980. Today, people in Italy have a life expectancy at birth which is around 5 years longer than the one measured in the US population (Figure 2A). To study the impact of income inequality on health in the USA and Italy, we analyze two surveys that combine information on income and health for the same individuals. While extensive longitudinal data are available for the USA, only cross-sectional data are available for Italy (see Section 2.1 for details). Hence, we compared the health characteristics of individuals with different incomes from 1999 to 2021 in the case of USA and only for the year 2019 in the case of Italy. For the USA, we also considered the special case of the households that where in the bottom quintile of the income distribution in the year 1999 and remained there in later years. In the following, we refer to these households as persisters.

We first considered the general health status of the surveyed populations and computed the fraction of households where the reference person had fair or poor health (scored 4–5 on a scale from 1 to 5). We divided people depending on their income considering those belonging to the top 20% and the bottom 20% of the distribution and found that in the USA health status was strongly dependent on income: Only <10% of people in the upper income quintile had fair/poor health while this percentage was 30% in 1999 for the people in the bottom income quintile (Figure 2B). The situation deteriorated since in 2019 around 40% of the people belonging to the bottom 20% had fair/poor health. The health status of persisters was even worst, with more than 50% claiming fair or poor health in 2019. The situation appears to be radically different in Italy, where the percentage of people with fair or poor health was <10% in 2019 with a very weak dependence on the income quintile (Figure 2C).

**Figure 3:**
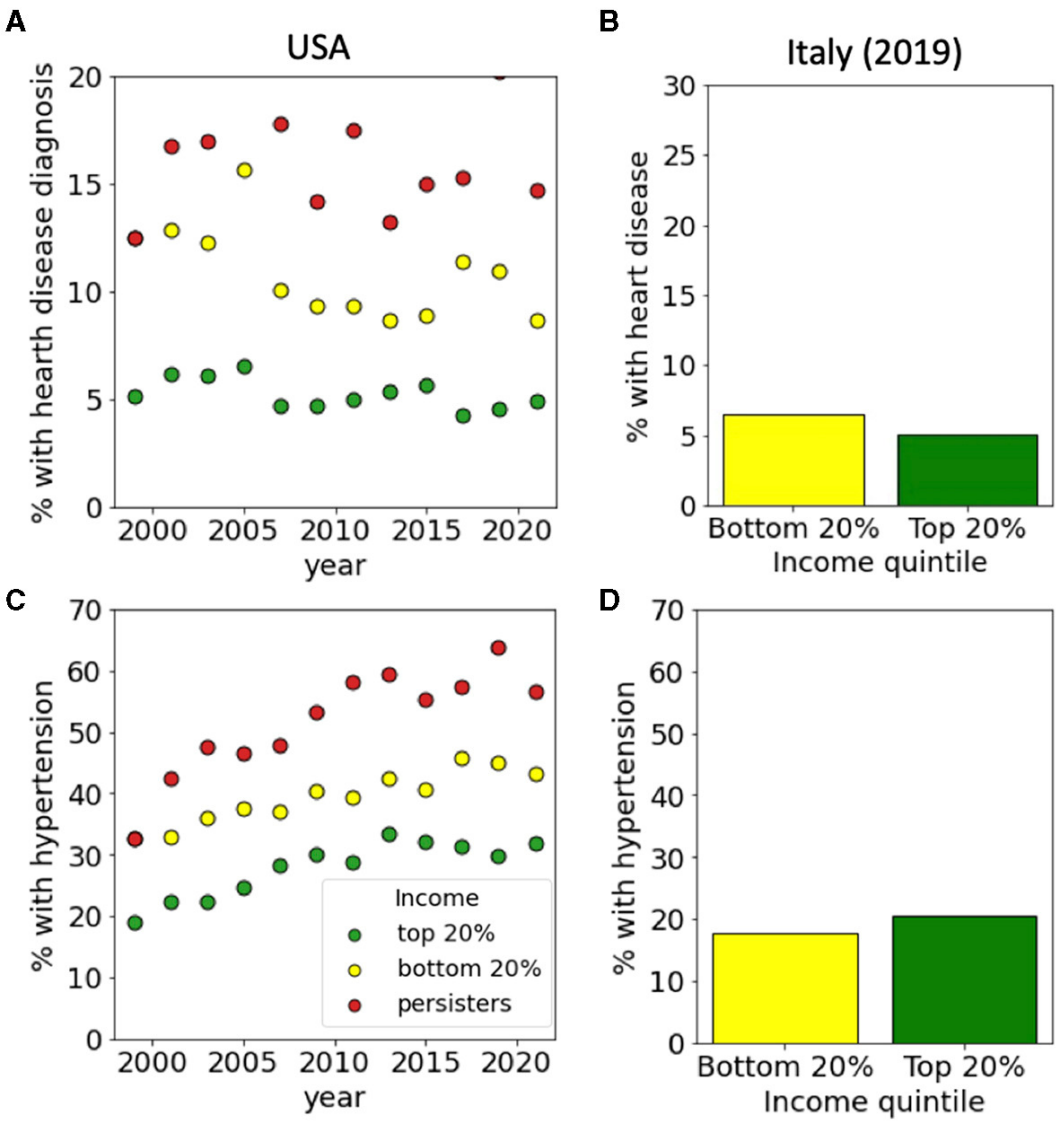
Income-dependent incidence of hearth disease, hypertension and diabetes. For the USA, incidences are reported for top and bottom income quintiles and for persisters from 1999 to 2021. For Italy, data is reported for top and bottom income quintiles in 2019. Panels refer to **(A, B)** heath disease, **(C, D)** hypertension for USA and Italy, respectively. Data sources: **(A, C)** PSID and **(B, D)** ISTAT-EIHS.

**Figure 4:**
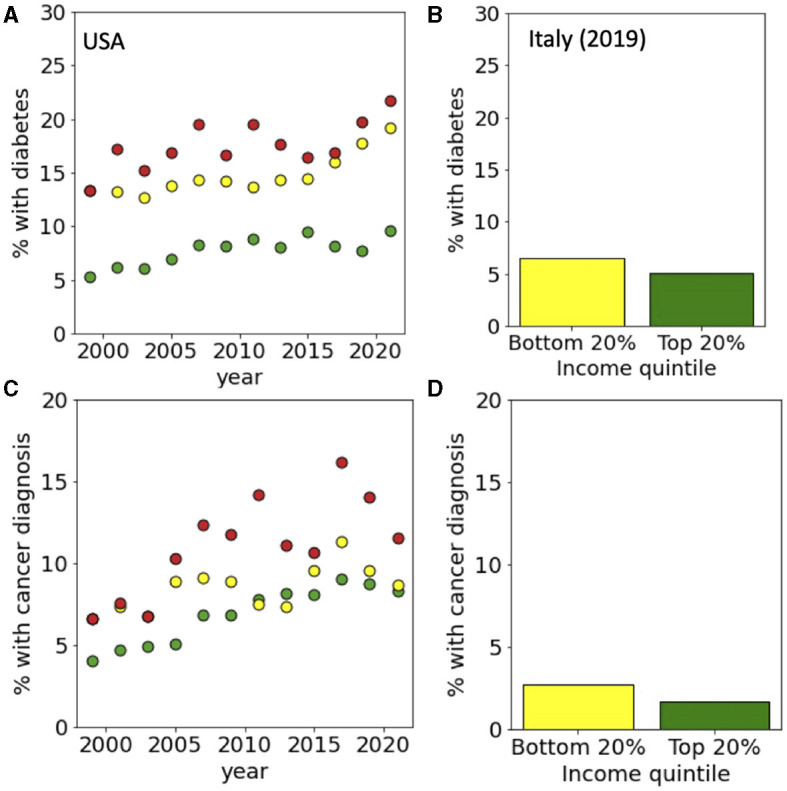
Income-dependent incidence of diabetes and cancer. For the USA, incidences are reported for top and bottom income quintiles and for persisters from 1999 to 2021. For Italy, data is reported for top and bottom income quintiles in 2019. Panels refer to **(A, B)** diabetes and **(C, D)** cancer for USA and Italy, respectively. Data sources: **(A, C)** PSID and **(B, D)** ISTAT-EIHS.

**Figure 5:**
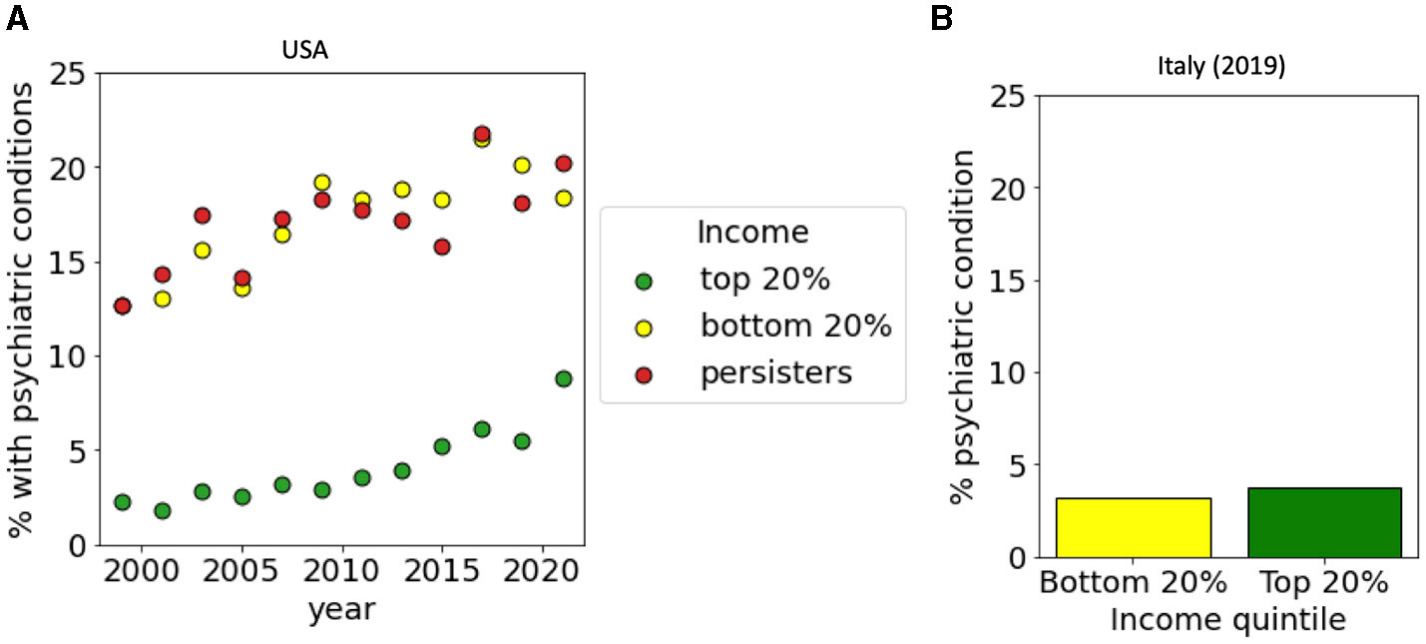
Income-dependent incidence of psychiatric conditions. **(A)** For the USA, incidences are reported for top and bottom income quintiles and for persisters from 1999 to 2021. **(B)** For Italy, data is reported for top and bottom income quintiles in 2019. Data sources: **(A)** PSID and **(B)** ISTAT-EIHS.

We then repeated the same analysis for a set of specific pathological conditions, starting from hearth disease which is the leading cause of death in the western world. Data from the USA, show that while only around 5% of people in the top 20% of the income distribution had a heart disease diagnosis, this percentage raised to 10% for people the bottom 20% and to 15% for persisters (Figure 3A). In Italy, we found instead that only around 5% of the people were diagnosed with heath disease with a small dependence on their income (Figure 3B). A similar trend is seen for hypertension (Figures 3C, D), diabetes (Figures 4A, B) and cancer (Figures 4C, D) whose incidence in the USA is increasing in time in a way that is dependent on income and persistence while in Italy the incidence is smaller and only weakly dependent on income. Finally, we considered people with psychiatric conditions in the USA and in Italy (Figure 5). We notice that the presence of psychiatric conditions strongly depend on income in the USA but not in Italy.

### 3.3 Obesity and food insecurity

**Figure 6:**
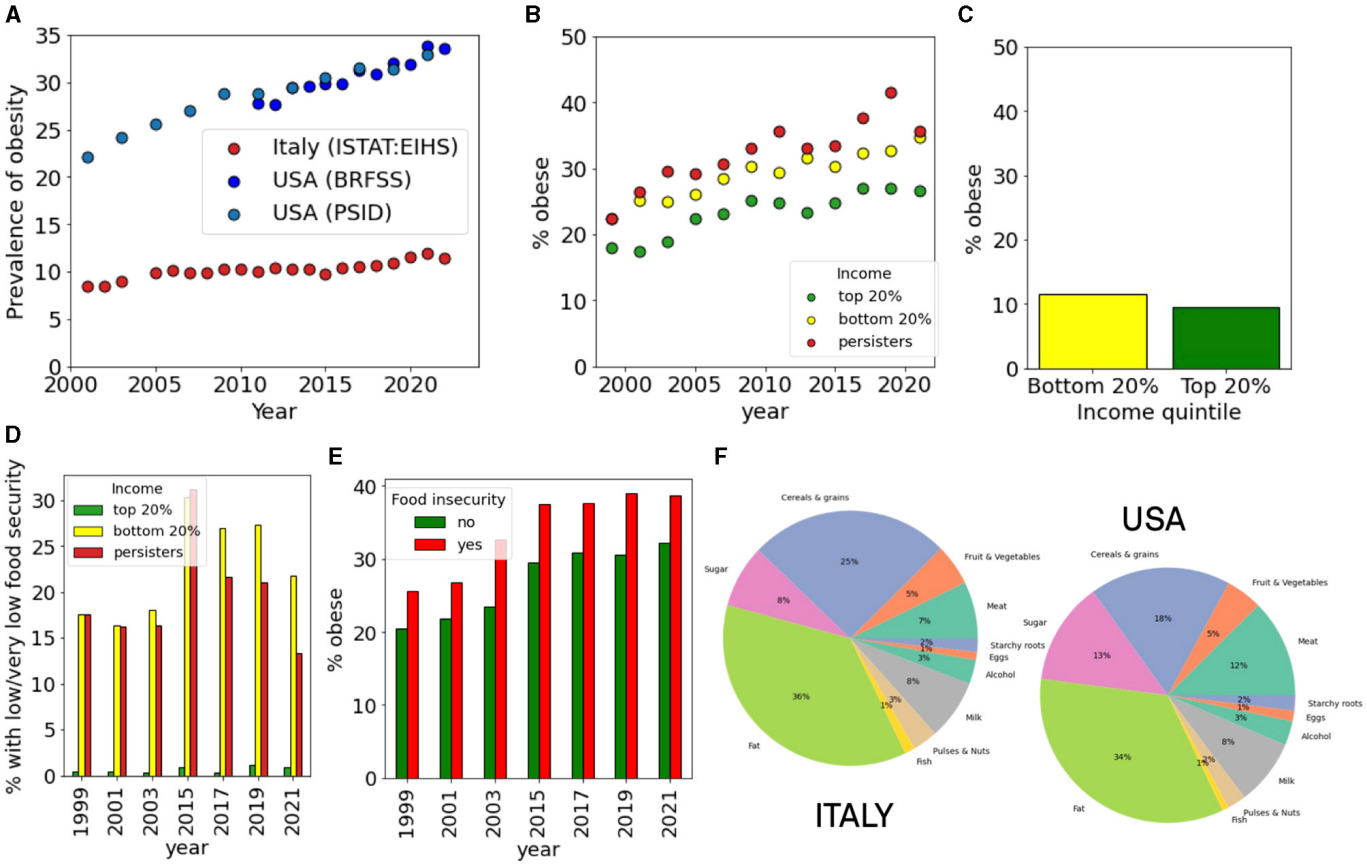
Food insecurity and obesity. **(A)** Incidence of obesity in the USA and Italy. **(B, C)** Income-dependent incidence of obesity in the **(B)** USA and **(C)** Italy. **(D)** Incidence of food insecurity for top and bottom income quintiles and for persisters in the USA. **(E)** Incidence of obesity in households with or without food insecurity in the USA. **(F)** The dietary composition in Italy and in the USA.

Obesity is an important risk factor for all the pathological conditions analyzed above. Its incidence has been increasing in all the western countries, including Italy and the USA where the increase has been particularly striking, approaching 35% of the entire population (Figure 6A). When we distinguished between income classes, we observed that the increase is more marked in the lower quintile of the income distribution than in the top one. Persisters displayed an even larger incidence of obesity, reaching up to 40% (Figure 6B). In Italy, obesity is increasing but the level is still not comparable with the USA. Also in this case, we observed a dependence on income, with lower income groups displaying larger incidence (Figure 6C). We then analyzed food insecurity in the USA and observed that it is largest among persisters (Figure 6D). Food insecurity was also correlated with obesity, since households experiencing food insecurity had also a larger incidence of obesity (Figure 6E). The differences in obesity could be associated to the dietary patterns in USA and Italy (Figure 6F). By analyzing the composition of food products available in Italy and USA, we noticed marked differences in the amount of sugar and meat which is larger in the USA, while the fraction of cereal and grains is larger in Italy. Furthermore, the caloric intake *per capita* is generally larger in the USA than in Italy.

### 3.4 Comorbidity patterns

**Figure 7:**
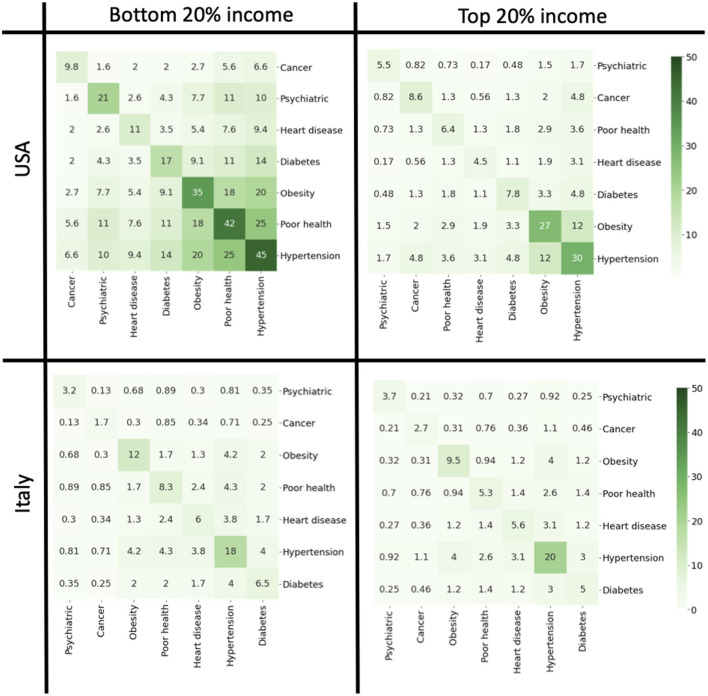
Comorbidity matrices for the top and bottom quintiles for USA and Italy in 2019. The off-diagonal elements of the matrix report the probabilities that a person is affected by both conditions. The diagonal elements indicate the incidence of each pathology.

**Figure 8:**
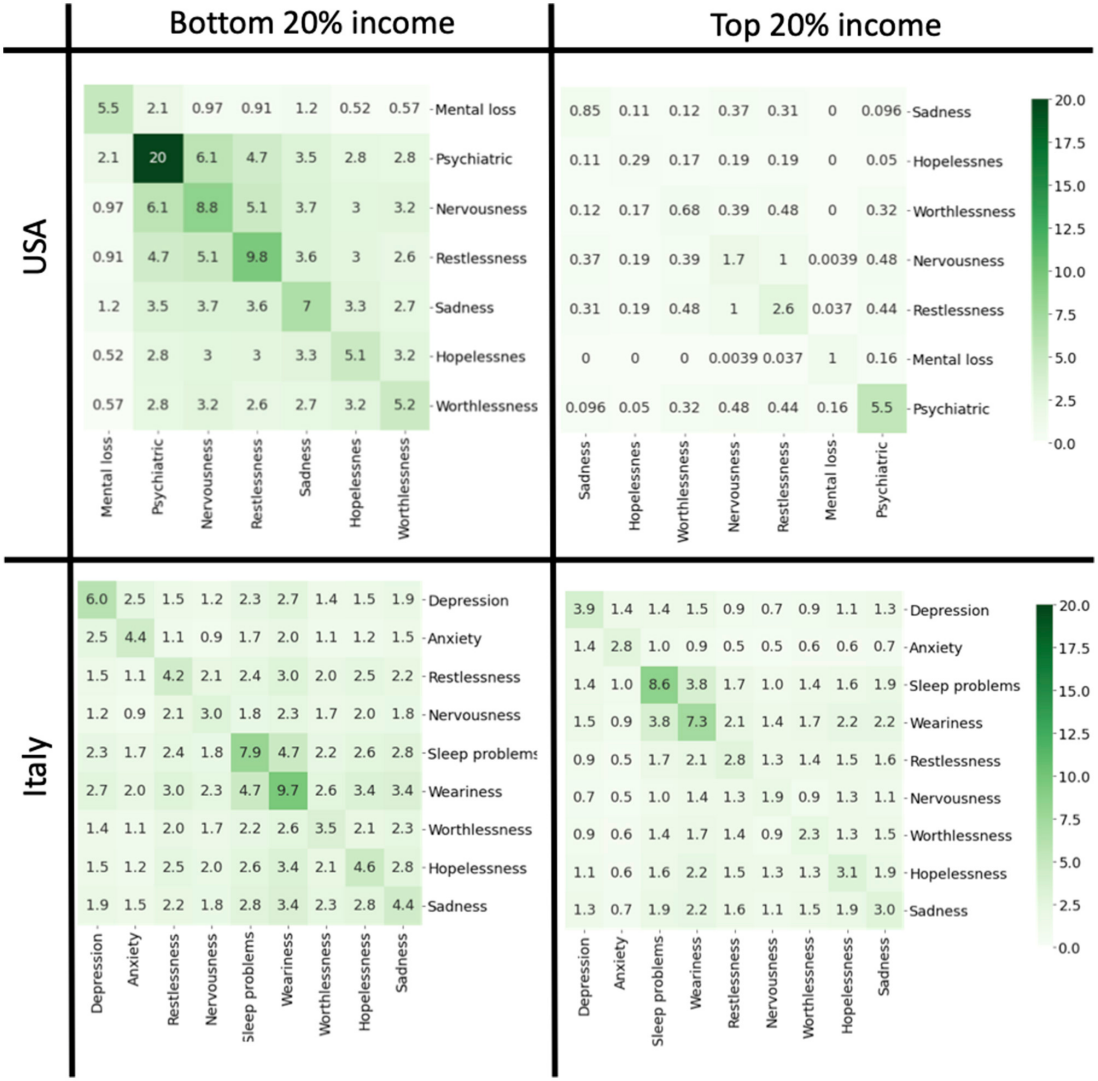
Comorbidity matrices relative to mental health and depression for the top and bottom quintiles for USA and Italy in 2019. The off-diagonal elements of the matrix report the probabilities that a person is affected by both conditions. The diagonal elements indicate the incidence of each condition. Data sources: PSID (USA) and ISTAT-EIHS (Italy).

Survey data allowed us to estimate patterns of comorbidity and their dependence on income. We considered the set of pathological conditions (cancer, diabetes, poor health, hypertension, heart disease, obesity, poor healt, and psychiatric problems) and constructed comorbidity matrices for people belonging to the bottom and top quintile of the income distribution. The matrix element *M*_*ij*_ describes the probability that a subject has both pathologies *i* and *j*. The diagonal matrix *M*_*ii*_ represents the incidence of the pathology *i* in the population. We then performed hierarchical clustering of the matrices to visualize groups of pathologies that are like to occur together in the same subject. In the USA, we observed very different comorbidity patterns between the top and bottom income classes (Figure 7). In the bottom 20% income class, we noticed a cluster related to hypertension, poor health, obesity and diabetes which is considerably attenuated in the top 20% income class. Comorbidity was instead less present in Italy where co-occurrence of different pathologies was scarce with little dependence on income. We then analyzed with a similar strategy the co-occurrence of psychiatric conditions and symptoms associated to depression (Figure 8). Also in this case, we observed clusters of co-occurrence in subjects from low income families from the USA, while the patterns essentially disappeared for people with high income. In Italy, on the other hands, the patterns are are similar for low and high income people.

## 4 Discussion

An extensive literature has shown that wealth and income inequalities have increased worldwide in the past decades (20–22) due to tax policies, financial regulations and the deterioration of welfare programs in many countries (23, 24). The widening of wealth and income inequality are also associated with increased levels of social immobility observed in many developed economies (25, 26). In this paper, we explored the relations between income inequality health by comparing longitudinal survey data from Italy and USA.

Life expectancy at birth in Italy and the USA in 1960 was the same but data shows that it started to diverge from the 1980's onwards. Although the USA is one of the richest countries in the world, its population has a lower life expectancy at birth than the Italians. This simple observation raises many questions: why have the two countries diverged since the 1980's? Is this fact related to differences in health and medical care expenditure? What makes the population in the USA less healthy than in Italy, where life expectancy is longer and thus in general the quality of life better? The comparison of a longitudinal study of American households and the cross-sectional data available for Italy related to health allowed us to identify a fraction of the population that remained persistently at the bottom of the income distribution, that we denoted as persisters. In the USA, this population displayed the worst situation from the point of view of health. In Italy, on the other hand, the health status of the poorest segment is much better when compared to the USA. Many factors could explain this, but one of them may be the presence of a universal national health system, which allows to the poorest segments of the population to access healthcare.

It is interesting to look closely at the diseases that affected the low income population in the USA. Hearth disease is the first cause of death in the USA and worldwide. Our analysis shows very clearly that the incidence of this pathology is not equally distributed throughout the population: the persisters are significantly more affected than the rest of the population. Similarly, our analysis also shows that hypertension, diabetes and cancer are unequally distributed across the population. These diseases are not equally distributed not only in USA where there is a great inequality in wealth but also in in low- and middle-income countries (LMICs) both in adults and adolescents (27–29).

One important risk factor for these diseases is related to a nutritionally poor diet. It was recently shown through a meta-analysis that ultra-processed food consumption is associated with body fat during childhood and adolescence (30). The main reasons of this kind of association is due by the fact that processed food contains a high quantity of calories, often contain excess refined sugar, saturated and trans fat, and a lot of preservatives (31, 32). Moreover, most processed food contains highly refined carbohydrates that alter insulin physiology and promote adipose tissue deposition (33, 34). Recent studies showed that extremely processed food containing extra lipid substances and sugar-sweetened beverages change neurobiological reward pathways involved in eating behaviors, encouraging food cravings and excessive food intake (35, 36). A review of the recent literature shows that in the USA the energy intake from ultra-processed food is higher than 50% of the total, representing the highest value worldwide, while in Italy the level is around 10% (37).

In the present day, food insecurity is not only a problem in developing countries but also in developed countries. As our results show, levels of food insecurity in the USA are much higher than in Italy, and are particularly prevalent in extremely poor people. The connection with ultra-processed food is therefore related to their precarious economic conditions (38). The lower incidence of food insecurity and obesity in Italy may be related to the different diet between the two countries: the Western diet in USA and a Mediterranean diet in Italy. It is well-reported the protective effect of the Mediterranean diet toward obesity with a higher consumption of vegetables and less consumption of sugar and red meat (39, 40). Our analysis of the composition of the food supply confirms this showing a larger consumption of meat and sugar in the USA with respect to Italy.

Psychiatric problems, changes related to the mood, such as sadness, hopelessness, worthlessness, nervousness are also more present in low income population in the USA. These conditions are often connected to food insecurity as well as other problems, such alcohol and drugs abuse (41). Interestingly, in Italy the mental problems are more related to depression, while sleep problems and weariness are equally present in the population independently on the income level. This is probably due to structure of Italian society where loneliness is often compensated by solidarity. Furthermore, conditions of mental stress appears to be equally distributed across the income distribution in Italy but not in the USA.

Comorbidity refers to the presence of different pathological conditions in the same person and is usually associated with with worse clinical outcome and higher costs for the healthcare services (42). We have compared comorbidity across the income distribution for Italy and USA and found a high probability for the co-occurrence of obesity, diabetes and hypertension in the US low income groups as opposed to the high income population where the probability is smaller. In Italy, on the other hand, comorbidity is much less prevalent in way that is largely independent on income.

In order to compare the situation in Italy and USA, we focused most of the analysis on the year 2019, since this is the most recent year for which comparable data are available for both countries. It is interesting to discuss what happened after the COVID-19 pandemic that started in 2020. We have analyzed data for the USA for the year 2021, which is right after the pandemic. While the effect of COVID-19 related mortality is evident in the life expectancy curves, we did not observe striking changes in the other health indicators from the year 2019 to the year 2021 in the USA. The only interesting effect is seen for psychiatric conditions: we observed that the incidence remained constant for low income groups, but for the high income group it raised from 5.5 to 8.9%.

Our study is based on the analysis survey data, which have several limitations. Bias in the data may arise for instance due to dropout, when participants of specific income group dropout at higher rate; self-reporting errors, when participants of given income group misrepresent their conditions; panel conditioning, where participants alter their behavior due to their awareness of being studied; under-representation of some ethnic groups. Despite these potential sources of bias, panel studies provide an extremely rich informative set of data.

## 5 Conclusion

In conclusions, our comparative analysis highlights the differences in health across the income distribution in Italy and USA. The striking differences between these two countries suggests specific policy interventions. It is fundamental importance to implement and maintain a national health system providing universal coverage and at the same time promote an healthy lifestyle that minimizes the consumption of ultra-processed food. These policies represent in our opinion an effective strategy to improve general health conditions and reduce health inequalities.

## Data availability statement

Publicly available datasets were analyzed in this study. The datasets analyzed for this study can be accessed as follows: Panel Study of Income Dynamics, public use dataset. Produced and distributed by the Survey Research Center, Institute for Social Research, University of Michigan, Ann Arbor, MI is available from: https://psidonline.isr.umich.edu/, Bank of Italy data are available at: https://www.bancaditalia.it/statistiche/basi-dati/rdc/index.html, World Bank data are available at: https://data.worldbank.org/, and ISTAT-EHIS data are available at: https://www.istat.it/en/archivio/214912.

## Author contributions

SZ: Conceptualization, Data curation, Formal analysis, Funding acquisition, Investigation, Methodology, Project administration, Resources, Software, Validation, Visualization, Writing – original draft, Writing – review & editing. CL: Conceptualization, Data curation, Formal analysis, Funding acquisition, Investigation, Methodology, Project administration, Resources, Validation, Visualization, Writing – original draft, Writing – review & editing.
